# Histone Demethylase Retinoblastoma Binding Protein 2 is Overexpressed in Hepatocellular Carcinoma and Negatively Regulated by hsa-miR-212

**DOI:** 10.1371/journal.pone.0069784

**Published:** 2013-07-29

**Authors:** Xiuming Liang, Jiping Zeng, Lixiang Wang, Ming Fang, Qing Wang, Min Zhao, Xia Xu, Zhifang Liu, Wenjuan Li, Shili Liu, Han Yu, Jihui Jia, Chunyan Chen

**Affiliations:** 1 Department of Microbiology/Key Laboratory for Experimental Teratology of Chinese Ministry of Education, Shandong University School of Medicine, Jinan, P. R. China; 2 Department of Biochemistry, Shandong University School of Medicine, Jinan, P. R. China; 3 Department of Pharmacology, Shandong University School of Medicine, Jinan, P. R. China; 4 Department of Hematology, Qilu Hospital, Shandong University, Jinan, P. R. China; University of Hong Kong, Hong Kong

## Abstract

**Background:**

The H3K4 demethylase retinoblastoma binding protein 2 (RBP2) is involved in the pathogenesis of gastric cancer, but its role and regulation in hepatocellular carcinoma (HCC) is unknown. We determined the function of RBP2 and its regulation in HCC *in vitro* and in human tissues.

**Methods:**

We analyzed gene expression in 20 specimens each of human HCC and normal liver tissue by quantitative real-time PCR and immunohistochemistry. Proliferation was analyzed by foci formation and senescence by β-galactosidase staining. Promoter activity was detected by luciferase reporter assay.

**Results:**

The expression of RBP2 was stronger in cancerous than non-cancerous tissues, but that of its binding microRNA, *Homo sapiens* miR-212 (hsa-miR-212), showed an opposite pattern. SiRNA knockdown of RBP2 significantly upregulated cyclin-dependent kinase inhibitors (CDKIs), with suppression of HCC cell proliferation and induction of senescence. Overexpression of hsa-miR-212 suppressed RBP2 expression, with inhibited cell proliferation and induced cellular senescence, which coincided with upregulated CDKIs; with low hsa-miR-212 expression, CDKIs were downregulated in HCC tissue. Inhibition of hsa-miR-212 expression upregulated RBP2 expression. Luciferase reporter assay detected the direct binding of hsa-miR-212 to the RBP2 3′ UTR.

**Conclusions:**

RBP2 is overexpressed in HCC and negatively regulated by hsa-miR-212. The hsa-miR-212–RBP2–CDKI pathway may be important in the pathogenesis of HCC.

## Introduction

Hepatocellular carcinoma (HCC) is one of the most common tumors in China [1]–[3]. Its early diagnosis is difficult. HCC can be diagnosed effectively in some cases with serological assessment [4]–[6], but the molecular mechanism of cell proliferation and effective biological markers for diagnosis are unclear.

Retinoblastoma binding protein 2 (RBP2), a newly found histone demethylase, belongs to the JARID1 protein family [7]–[9] and was found to interact with pRb, regulating cellular differentiation and embryonic development [10]–[11]. RBP2 is a kind of H3K4 demethylase, contributing to the demethylation of H3K4me3 and H3K4me2 [12]–[14]. Recently, RBP2 was found to be associated with cancer. Upregulation of RBP2 was a characteristic of a drug-tolerant cancer cell subpopulation [15]. We found RBP2 overexpressed in gastric cancer, and its inhibition could trigger cancer-cell senescence [16]. We also found RBP2 overexpressed in HCC. RBP2 was significantly underexpressed in normal liver tissue but strongly expressed in cancerous liver tissues, so RBP2 may take part in the development of HCC. Furthermore, siRNA inhibition of RBP2 expression could decrease the proliferation and induce senescence of high-differentiated HepG-2 cells and low-differentiated SMMC-7721 cells, 2 representative HCC cell lines, but the mechanism of RBP2 overexpression has not been elucidated.

MicroRNAs (miRNAs), non-coding 20– to 23-nt RNAs, play a key role in gene expression in different kinds of cancer [17]–[18]. MiRNAs can bind to the seed sequence of the 3′ UTR of target genes and negatively regulate their expression in cell proliferation, differentiation, senescence, and apoptosis, for example. MiRNAs also participate in cancer metastasis and cancerous angiogenesis by regulating specific genes. *Homo sapiens* miRNA-212 (hsa-miR-212) has 2 sites binding in the 3′ UTR of RBP2. It is downregulated and suppresses methyl-CpG-binding protein MeCP2 in human gastric cancer [19]. Downregulation of hsa-miR-212 increasing the expression of HB-EGF may be involved in cetuximab resistance in head and neck squamous cell carcinoma [20].

In this research, we investigated the involvement of RBP2 in HCC and the miRNA implicated in its regulation in human HCC. RBP2 inhibition and hsa-miR-212 overexpression may lead to cell proliferation arrest and cellular senescence by depressing cyclin-dependent kinase inhibitors (CDKIs; p16^ink4a^, p21^CIP2^ and p27^kip1^). RBP2 and hsa-miR-212 may be biomarkers for diagnosis of HCC and therapeutic targets in its treatment.

## Materials and Methods

The ethics committee of Shandong University School of Medicine approved this study and all participants provided written informed consent.

### Clinical Samples

We obtained 20 samples of HCC from patients at Bengbu Medical University, AnHui Province, China, immediately after surgery and stored them in formalin. The diagnosis of HCC was confirmed by histological examination; no patient had undergone chemotherapy or radiotherapy before surgery. The general characteristics of patients are in **Table S1**. We excluded patients with a history of alcohol abuse.

### Other Materials and Methods

Immunohistochemistry, cell culture [21]–[23] and siRNA interference, RNA extraction, RT-PCR and real-time PCR (primers are in Table 1), plasmid transfection and luciferase reporter gene assay (primers and methods are in Table 2), protein extraction and western blot assay, clonal formation assay and senescence-associated β-galactosidase (SA-β-Gal) staining are described in **Data S1**.

**Table 1:** PCR primer sequences used in the study.

Genes	Primers
RBP2	5′-GCTGCTGCAGCCAAAGTTG-3′ (forward)
	5′-AGCATCTGCTAACTGGTC-3′ (reverse)
p16^ink4a^	5′-TTCCTGGACACGCTGGT-3′ (forward)
	5′-CAATCGGGGATGTCTGAG-3′ (reverse)
p21^CIP2^	5′-GCGACTGTGATGCGCTAAT-3′ (forward)
	5′-TAGGGCTTCCTCTTGGAGAA-3′ (reverse)
p27^kip1^	5′-ATGTCAAACGTGCGAGTGTCTAA-3′ (forward)
	5′-TTACGTTTGACGTCTTCTGAGG -3′ (reverse)
hsa-miR-212	5′ - GGATCC TGCGTTGATCAGCACC - 3′ (forward)
	5′- AAGCTTCCCCTCTGGGACATCTTT - 3′ (reverse)
3′ UTR of hsa-miR-212	5′ - ACTAGT GCAGATGCTGTTGAATAA -3′ (forward)
	5′ - AAGCTT CTACAAAGAAGGAATGG - 3′ (reverse)
hsa-miR-212 inhibitor	5′-GATCCCCATTGTCAGAGGTCAGTGCCGGTTTTTTGGAAG-3′(forward)
	5′-TCGACTTCCAAAAAACCGGCACTGACCTCTGACAATGGG-3′ (reverse)
β-actin	5′ - AGTTGCGTTACACCCTTTCTTG - 3′ (forward)
	5′ - CACCTTCACCGTTCCAGTTTT - 3′ (reverse)

**Table 2:** Primer sequences for construction of wild type (WT) and mutants (MUT1 and 2) of the 3′ UTR of retinoblastoma binding protein 2 (RBP2) by the overlap extension PCR method.

Category	Primers
Sequences for construction of WT RBP2 3′ UTR	5′-ACTAGTGCAGATGCTGTTGAATAA-3′ (UTR sense)
	5′-AAGCTTCTACAAAGAAGGAATGG-3′ (UTR antisense)
Sequences for construction of MUT1	5′-GTATAGGCTGTGAAGTAGTCAGAGACA-3′ (UTR MUT1 primer-1)
	5′-TGTCTCTGACTACTTCACAGCCTATAC-3′ (UTR MUT1 primer-2)
Sequences for construction of MUT2	5′-ATATGCTGCTGGATGACTGAAATATA-3′ (UTR MUT2 primer-1)
	5′-TATATTTCAGTCATCCAGCAGCATAT-3′ (UTR MUT1 primer-2)

### Statistical Analysis

Quantitative data are expressed as mean±SD or SEM. Student’s *t* test was used to analyze the differences between groups. P<0.05 was considered statistically significant.

## Results

RBP2 is overexpressed and hsa-miR-212 underexpressed in human HCC.

**Figure 1:**
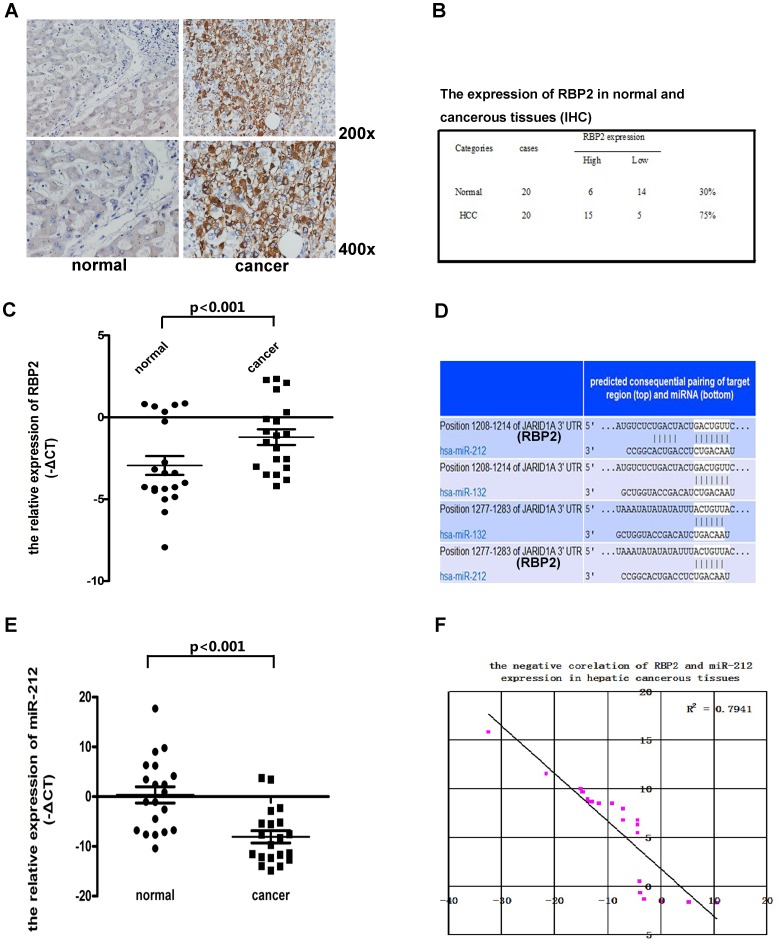
RBP2 was overexpressed and hsa-miR-212 underexpressed in human hepatic cancer tissues. (A) Representative immunostaining of RBP2 overexpressed in normal and cancer tissue. (B) Quantification of RBP2 expression in normal and cancerous tissues. (C and E) RBP2 and hsa-miR-212 expression in normal and hepatic cancerous tissues. Each point represents 1 sample. Horizontal medium bar is mean and whiskers are SD. (D) The predicted miRNA that can bind to the 3′ UTR of RBP2 and regulate its expression. (F) Linear plot of data from C and E showing a negative correlation between RBP2 and hsa-miR-212 expression in hepatic cancerous tissues. *P*<0.001.

Immunohistochemical staining revealed higher RBP2 protein expression in HCC than normal liver tissue **(** **Figure 1A** **)**, and the mRNA level of RBP2 was higher in HCC than normal tissue **(** *P*<0.001; **Figure 1C** **)** [24]. RBP2 was highly expressed in most HCC tissues (75%), whereas most normal tissues (70%) showed low RBP2 expression **(** **Figure 1B** **)**. Overexpression of RBP2 at the mRNA and protein levels in HCC tissue suggested that RBP2 might participate in the initiation and development of HCC.

Hsa-miR-212, the predicted miRNA that could bind to the 3′ UTR of RBP2 **(** **Figure 1D** **)**, may regulate its expression in these clinical samples. Hsa-miR-212 level was significantly lower in HCC than normal tissue **(**P<0.001; **Figure 1E** **)**, which suggested a negative regulation between RBP2 and hsa-miR-212 in HCC. RBP2 and miR212 levels were negatively correlated (R^2^ = 0.7941, **Figure 1F**).

### Inhibition of RBP2 Expression in HCC Cells Repressed Cell Proliferation and Induced Cell Senescence by Upregulating CDKIs

**Figure 2:**
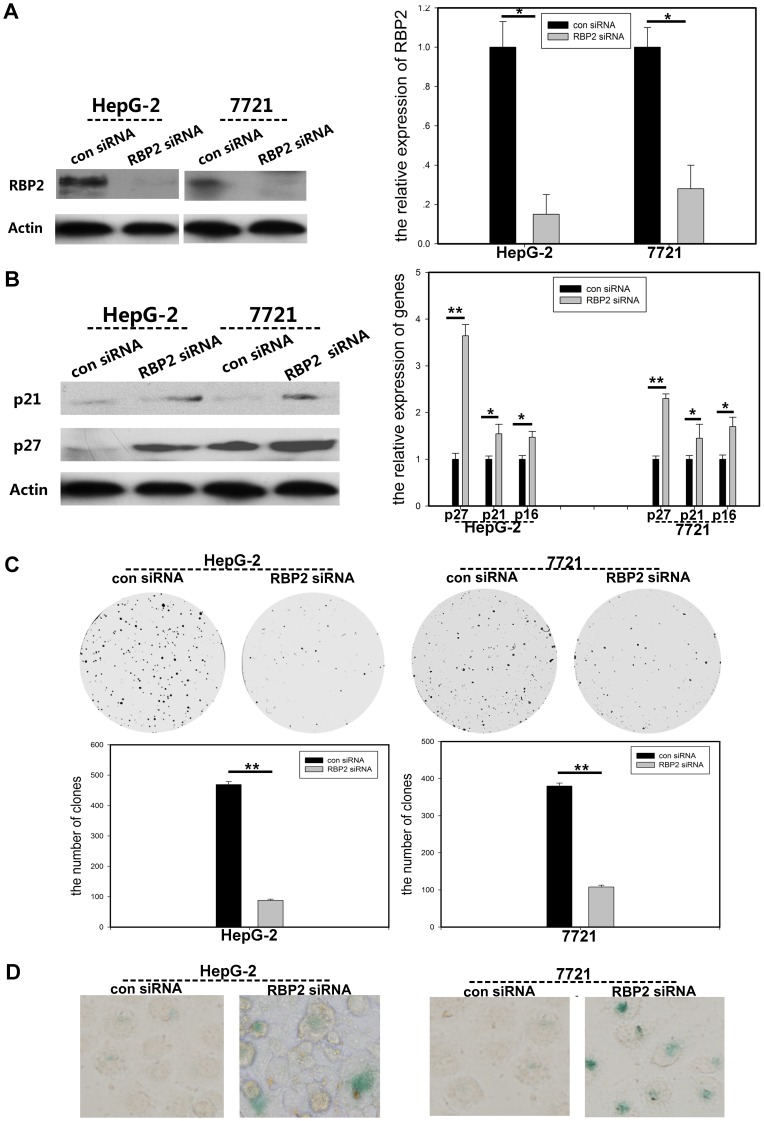
SiRNA knockdown of RBP2 suppressed HCC cell proliferation and induced cellular senescence by regulating cyclin-dependent kinase inhibitors (CDKIs). (A) Western blot analysis (left) and quantitative RT-PCR (qRT-PCR; right) of RBP2 protein and mRNA expression, respectively, with siRNA knockdown in HepG-2 and SMMC-7721 cancer cells. (B) Western blot analysis (left) and qRR-PCR analysis protein and mRNA expression of p16^ink4a^, p21^CIP2^ and p27^kip1^ with RBP2 siRNA knockdown. (C) Foci formation in cancer cells with RBP2 siRNA knockdown. (D) Cell senescence with RBP2 siRNA knockdown. Senescent cells were stained blue by SA-β-Gal staining. 400× magnification. Data are mean±SD of 3 biological replicates, * and **: *P*<0.05 and <0.01 compared with control.

Next, we investigated the mechanism by which RBP2 participates in hepatic oncogenesis in 2 hepatic cancer cell lines, HepG-2 and SMMC-7721 cells. The mRNA level of RBP2 was decreased with RBP2 siRNA knockdown, by about 80% and 72% in HepG-2 and SMMC-7721 cells, respectively **(** **Figure 2A** **; Figure S1A)**, and RBP2 protein level was decreased **(** **Figure 2A** **)**. At the same time, H3K4me2 and H3K4me3 expression was significantly increased with RBP2 depletion **(Figure S2C)**. The expression of known targets of RBP2, namely, the CDKIs p16^ink4a^, p21^CIP2^ and p27^kip1^, was markedly increased with RBP2 siRNA knockdown **(** **Figure 2B** **; Figure S2A)**. As a result, the proliferation of HCC cells was sharply restricted **(** **Figure 2C** **)**. Cell senescence was induced with RBP2 siRNA knockdown for 72 hr in cancer cells **(** **Figure 2D** **)**. Therefore, CDKI expression could be regulated by RBP2, and knocking down RBP2 expression could repress cell proliferation and induce cell senescence.

### Overexpression of RBP2 Blocked CDKI Expression *in vitro*

**Figure 3:**
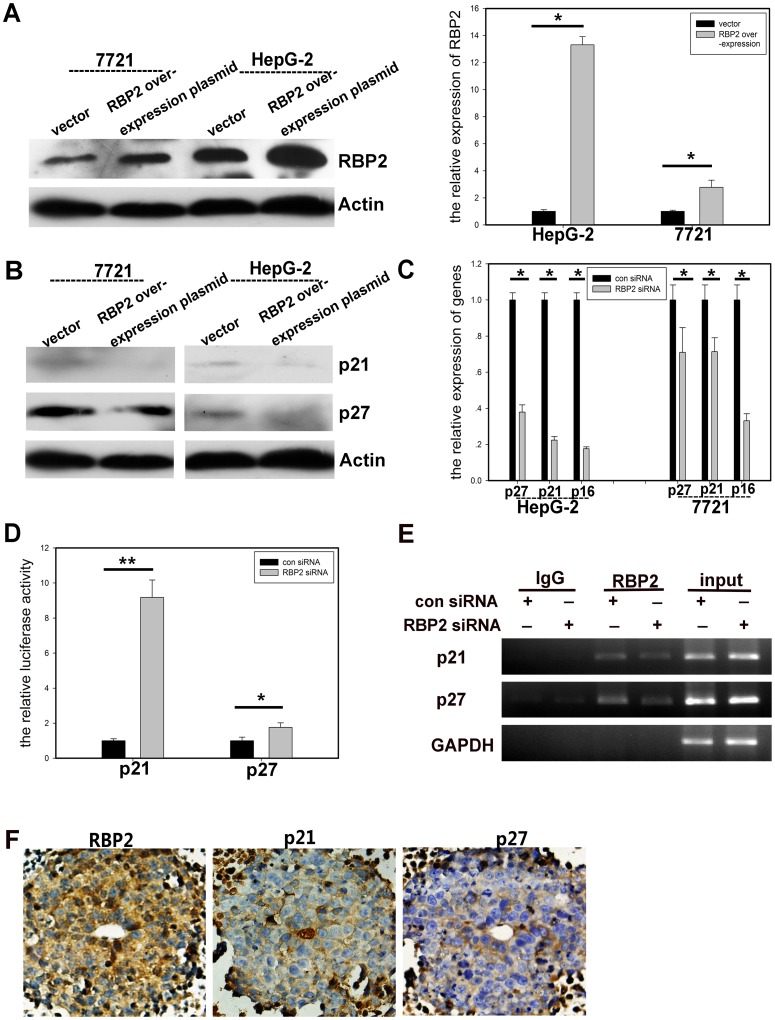
RBP2 overexpression inhibited the expression of CDKIs, and p21^CIP2^ and p27^kip1^ were directly regulated by RBP2. (A) Western blot analysis (left) and qRT-PCR analysis (right) of RBP2 protein and mRNA expression, respectively, in hepatic cancer cells with RBP2 overexpression. Western blot analysis (B) and qRT-PCR (C) of protein and mRNA expression of p16^ink4a^, p21^CIP2^ and p27^kip1^ with RBP2 overexpression. (D) P21^CIP2^ and p27^kip1^ promoter activity in RBP2 knocked-down HepG-2 cells. (E) ChIP assay of RBP2 directly binding to the promoters of p21^CIP2^ and p27^kip1^. (F) Immunohistochemical staining of co-localization of RBP2, p21^CIP2^, and p27^kip1^ in representative serial sections of tumors formed by injecting HepG-2 cells into nude mice. 600× magnification. Data are mean±SD of 3 biological replicates, * and **: *P*<0.05 and <0.01 compared with control.

RBP2 mRNA and protein expression was induced with an overexpression plasmid **(** **Figure 3A** **; Figure S1B)** and the expression of the CDKIs p16^ink4a^, p21^CIP2^ and p27^kip1^ was downregulated **(** **Figure 3B and C** **; Figure S2B)**.

### Expression of the CDKIs p21^CIP2^ and p27^kip1^ was Directly Regulated by RBP2

To determine whether p21^CIP2^ and p27^kip1^ were directly regulated by RBP2, siRNA knockdown of RBP2 expression greatly increased luciferase activity of p21^CIP2^ and slightly but significantly increased activity of p27^kip1^ in HepG-2 cells **(** **Figure 3D** **)**; ChIP assay confirmed the results **(** **Figure 3E** **)**. Therefore, RBP2 could directly bind to p21^CIP2^ and p27^kip1^ promoters and suppress their expression.

HepG-2 cells were injected into nude mice and tumors were formed **(Figure S1F)**. P21^CIP2^ and p27^kip1^ co-located with RBP2 on immunohistochemical staining of serial tissue sections of tumors: with RBP2 overexpression, the expression of p21^CIP2^ and p27^kip1^ was relatively low **(** **Figure 3F** **)**. Thus, RBP2 regulated the expression of CDKIs and affected cell proliferation, thereby contributing to the development of HCC in humans.

### RBP2 was Negatively Regulated by hsa-miR-212

**Figure 4:**
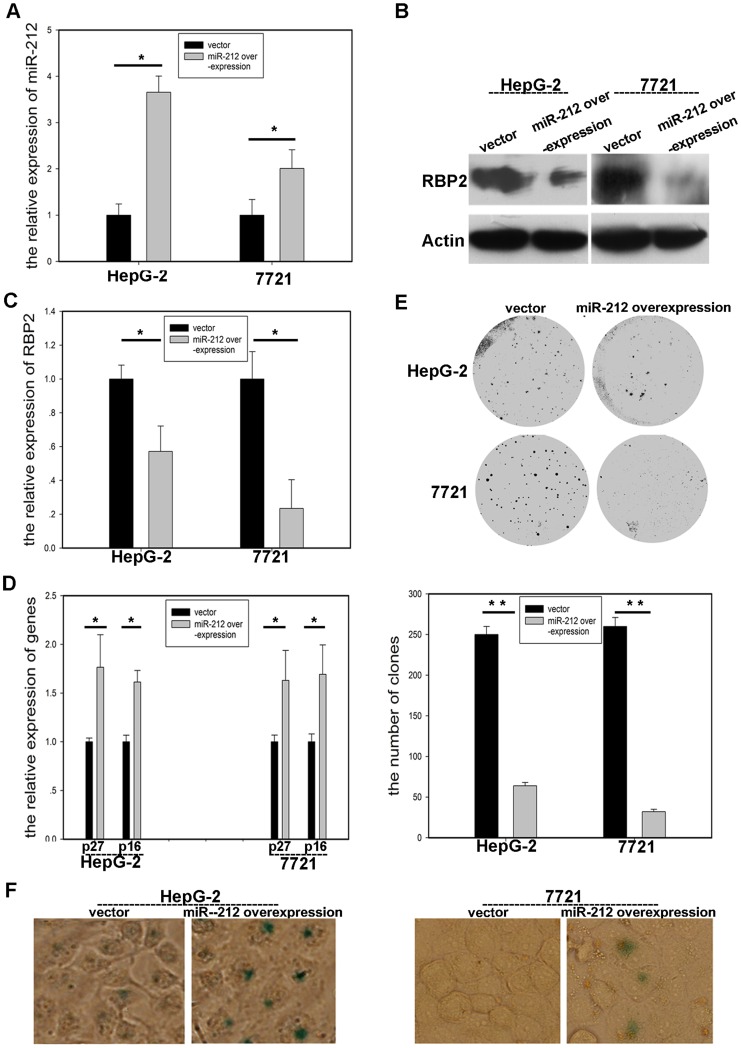
RBP2 expression was suppressed with hsa-miR-212 overexpression. (A) qRT-PCR analysis of Hsa-miR-212 mRNA expression in cancer cells with hsa-miR-212 overexpression. Western blot (B) and qRT-PCR analysis (C) of RBP2 protein and mRNA expression, respectively. (D) qRT-PCR analysis of mRNA expression of p16^ink4a^ and p27^kip1^, (E) foci formation (upper) and quantification (lower), and (F) cell senescence. 400× magnification. Data are mean±SD of 3 biological replicates, * *P*<0.05 compared with negative control.

**Figure 5:**
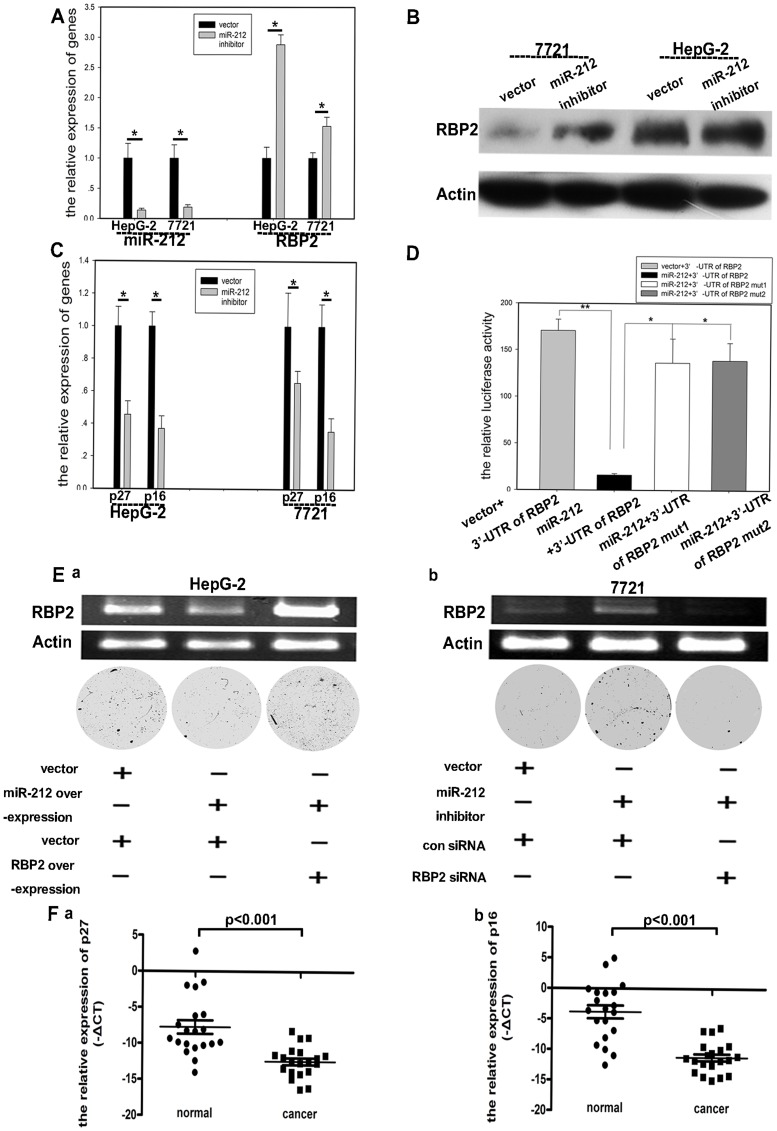
RBP2 expression was induced with hsa-miR-212 inhibition in HCC cell lines, and reduced p16^ink4a^ and p27^kip1^ expression was confirmed by qRT-PCR method in HCC with downregulated hsa-miR-212 expression. Effect of hsa-miR-212 inhibition on (A) qRT-PCR analysis of mRNA expression of Hsa-miR-212 and RBP2, (B) western blot analysis of protein expression of RBP2, and (C) qRT-PCR analysis of mRNA expression of p16^ink4a^ and p27^kip1^. (D) Luciferase activity of RBP2 3′ UTR with different treatment in SMMC-7721 cells. (E) RBP2 expression negatively regulated by hsa-miR-212 transfection and effect on HCC cell proliferation by foci formation. (F) qRT-PCR analysis of mRNA expression of p27^kip1^ and p16^ink4a^ in HCC tissues. Each point represents 1 sample. Horizontal medium bar is mean and whiskers are SD. Data are mean±SD of 3 biological replicates, * *P*<0.05 compared with control.

We confirmed that RBP2 was overexpressed in HCC and investigated the mechanism of the increased RBP2 expression. We found that RBP2 is a target of hsa-miR-212, and hsa-miR-212 could bind with 2 regions of the 3′ UTR of RBP2 **(** **Figure 1D** **)**. With hsa-miR-212 overexpression in HepG-2 and SMMC-7721 cells, hsa-miR-212 expression was significantly increased, which confirmed the success of hsa-miR-212 overexpression **(** **Figure 4A** **)**. RBP2 expression was markedly repressed with hsa-miR-212 overexpression **(** **Figure 4B and C** **; Figure S1C)**. The expression of p16^ink4a^ and p27^kip1^, targets of RBP2, was increased in the 2 cell lines **(** **Figure 4D** **)**, which agreed with previous findings. Accordingly, cell proliferation was repressed and cell senescence induced with hsa-miR-212 overexpression **(** **Figure 4E and F** **)**. To further confirm that RBP2 was negatively regulated by hsa-miR-212, transfection of hsa-miR-212 inhibitor plasmid in the 2 cell lines decreased the expression of hsa-miR-212 **(** **Figure 5A** **)** and increased that of RBP2 **(** **Figure 5A and B** **; Figure S1D)**. Accordingly, the expression of p16^ink4a^ and p27^kip1^ was decreased with hsa-miR-212 inhibition **(** **Figure 5C** **)**.

To ascertain whether hsa-miR-212 directly regulated the expression of RBP2, use of an RBP2 3′-UTR reporter vector significantly decreased luciferase activity with hsa-miR-212 overexpression in SMMC-7721 cells. However, co-transfection of hsa-miR-212 with the 2 mutants of the RBP2 3′ UTR reporter vector (mutation of the 2 binding sites of RBP2, **Table 2**) reversed the luciferase activity **(** **Figure 5D** **)**. Thus, RBP2 was a direct target of hsa-miR-212.

To confirm that RBP2 was negatively regulated by hsa-miR-212 and could affect cell proliferation, HepG-2 cells with hsa-miR-212 overexpression showed decreased RBP2 expression, with decreased cell proliferation **(** **Figure 5E** **, left)**. With RBP2 overexpression, RBP2 expression recovered accordingly, for enhanced cell proliferation **(** **Figure 5E** **, left)**. SMMC-7721 cells with hsa-miR-212 inhibitor showed increased RBP2 expression, with enhanced cell proliferation **(** **Figure 5E** **, right)**. RBP2 siRNA knockdown decreased RBP2 expression, for reduced cell proliferation **(** **Figure 5E** **, right)**. Furthermore, the expression of p16^ink4a^ and p27^kip1^ was downregulated in HCC tissues with inhibited hsa-miR-212 expression **(**both P<0.001; **Figure 5F** **)**. Therefore, low hsa-miR-212 expression resulted in overexpressed RBP2, which contributed to downregulation of p16^ink4a^ and p27^kip1^, thus confirming the hsa-miR-212–RBP2–CDKI pathway involved in HCC.

## Discussion

RBP2 is involved in the pathogenesis of gastric cancer, but its role and regulation in HCC was unknown. We determined the function of RBP2 and its regulation in HCC *in vivo* and *in vitro*. The expression of RBP2 was stronger in cancerous than non-cancerous human tissues, but that of its binding miRNA, hsa-miR-212, was reversed. SiRNA knockdown of RBP2 significantly upregulated CDKIs, with suppression of HCC cell proliferation and induction of cell senescence. Overexpression of hsa-miR-212 had a similar effect. Conversely, inhibition of hsa-miR-212 expression upregulated RBP2 expression. We found that RBP2 is overexpressed in HCC and negatively regulated by hsa-miR-212. The hsa-miR-212–RBP2–CDKI pathway may be important in the pathogenesis of HCC.

Epigenetic modulation participates in various biological processes by affecting chromatin stability and transcriptional repression or activation of target genes. Epigenetic molecules may play an important role in many types of tumorigenesis [25]. An important histone demethylase family, the JARID1 protein family, has been investigated widely in the biological process of higher organisms [18]. JARID1B/RBP2–H1/KDM5B was found to be a transcriptional regulator with tumor-suppressive potential in melamoma cells [26] and was used as a biomarker for a small subpopulation of slow-cycling melanoma cells [27]. The von Hippel-Lindau tumor-suppressor protein regulates gene expression and tumor growth via the histone demethylase JARID1C/KDM5C/SMCX in clear-cell renal cell carcinoma [28]. The expression pattern of JARID1D/KDM5D/SMCY significantly differed in meningiomas of male and female patients and contributes to the female predominance of this tumor [29].

RBP2, a member of the JARID family protein, also named JARID1A or KDM5A, has histone demethylase activity that could demethylate H3K4me3 and H3K4me2 significantly [8], [12]. Thus, RBP2 may affect the stability of chromatin through histone modulation. We first investigated the expression of RBP2 in human HCC and found the same expression as in gastric cancer. Recently, upregulation of RBP2/KDM5A expression was seen as characteristic of a drug-tolerant cancer cell subpopulation, cancer stem cells [15], which highlights the role of RBP2 in tumors and suggests its role in the initiation and development of HCC, but its mechanism in cancer was not eluciated.

MiRNAs affect tumorigenesis [30]–[32]. They can negatively regulate oncogenes and play a role as tumour suppressor genes but can also target tumour-suppressor genes and play a role as oncogenes. MiR-21 is overexpressed in most types of cancer [33]–[34] and Let-7 has an exclusively strong effect in lung cancer development [35]. Here, we found that RBP2 is a direct target of hsa-miR-212, and overexpressed RBP2 in HCC tissues may result from downregulated hsa-miR-212 expression. Hsa-miR-212 has been widely investigated [36]–[39]. We found that Hsa-miR-212 directly binds to the 3′ UTR of RBP2 and suppressed its expression. The suppressed RBP2 expression led to inhibited proliferation and induced senescence of hepatocellular cells by upregulating CDKIs. So, the low expression of hsa-miR-212 in HCC may result in overexpression of RBP2 and downregulation of RBP2 targets such as CDKIs, accompanied by cell proliferation and blocking cellular senescence in HCC, which contributes to its pathogenesis. Here, we provide more evidence of epigenetic molecule regulation by miRNAs.

However, the mechanism by which hsa-miR-212 is downregulated in HCC is not known. Infection with hepatitis B virus (HBV) may play a role in this process because HCC can result from HBV infection. As well, downregulation of hsa-miR-212 may be due to hypermethylation of DNA in the hsa-miR-212 promoter because downregulation of hsa-miR-212 expression is through DNA hypermethylation in human gastric cancer [40]. Recently, miR-212 expression was found to be deregulated in lung cancer [41]. However, other mechanisms may lead to the suppression of hsa-miR-212 expression in HCC. Also, downregulated hsa-miR-212 expression may not be the only mechanism leading to the overexpression of RBP2. We found that depletion of one miRNA, hsa-miR-212, antagonized the suppression of RBP2, which led to RBP2 overexpression in HCC, but further investigation is needed to elucidate the reason for the overexpressed RBP2 in HCC. Induced epigenetic molecules could also regulate the expression of miRNAs [42]–[44], but we found no evidence of induced RBP2 affecting hsa-miR-212 expression because siRNA knockdown of RBP2 did not change the expression of hsa-miR-212 **(Figure S1E)**.

In conclusion, our findings suggest that overexpressed RBP2 in HCC resulted from downregulated hsa-miR-212 expression and that overexpressed RBP2 could promote cell proliferation and suppress cell senescence by downregulating p16^ink4a^, p21^CIP2^ and p27^kip1^. The hsa-miR-212–RBP2–CDKI pathway in HCC may contribute to its initiation and development. Low expression of hsa-miR-212 and overexpression of RBP2 may be prognostic markers for HCC. As well, RBP2 and hsa-miR-212 expression may be a therapeutic target for HCC.

## Supporting Information

Data S1 Supporting Materials and Methods.
(DOC)

Figure S1 RBP2 expression was inhibited with transfection of RBP2 siRNA or enhanced with transfection of RBP2 overexpression plasmid in HCC cell lines and RBP2 was negatively regulated by hsa-miR-212 (A–B) RT-PCR analysis of the mRNA expression of RBP2 with transfection of RBP2 siRNA or RBP2 overexpression plasmid. (C–D) RT-PCR analysis of mRNA expression of RBP2 expression with transfection of hsa-miR-212 overexpression or inhibitor plasmid. (E) qRT-PCR analysis of mRNA expression of hsa-miR-212 with transfection of control or RBP2 siRNA. Data are mean±SD of biological replicates. (F) Confirmation of tumor development in mice with injection of HepG-2 cells.
(TIF)

Figure S2 p16 was negatively regulated by RBP2 and H3K4me2 and H3K4me3 expression was significantly increased with RBP2 knockdown in HCC cell lines. (A) Western blot analysis of protein expression of p16 with RBP2 suppression and (B) RBP2 overexpression. (C) Expression of H3K4me2 and H3K4me3 expression in HCC cell lines with RBP2 knockdown.
(TIF)

Table S1 General characteristic of the patients.
(DOC)
