# Supplementary material for: Histone Demethylase Retinoblastoma Binding Protein 2 is Overexpressed in Hepatocellular Carcinoma and Negatively Regulated by hsa-miR-212
Source: PLoS One. 2013 Jul 29;8(7):e69784. doi: 10.1371/journal.pone.0069784 (PMC3726779; doi:10.1371/journal.pone.0069784)
Supplement: Data S1 — Supporting Materials and Methods. (DOC) [file pone.0069784.s001.doc]

**2.4 Immunohistochemistry**

Tissue samples were obtained from tumors formed in nude mice 3 weeks after HepG-2 cell injection (1×10^6^ cells per mouse) and histochemical sections were created. Tissue sample sections from patients and controls were deparaffinized and dehydrated. After antigen retrieval, blocking endogenous peroxidase activity, and goat serum treatment, slides were incubated with the monoclonal antibody rabbit anti-human RBP2 (Sigma, USA; 1:150) or mouse anti-human p21^CIP2^ or p27^kip1^ (Santa Cruz Biotechnology, USA; 1:150) overnight at 4°C, then secondary antibody. Antibody binding was detected by the avidin-biotin-peroxidase method with DAB staining (Vector Laboratories, Burlingame, CA, USA).

**2.5 Cell culture and siRNA interference**

HepG-2 and SMMC-7721 cells were grown in a 5% CO_2_ atmosphere at 37°C in DMEM (Gibco, USA) and RPMI 1640 medium (Gibco, USA), respectively, supplemented with 10% fetal bovine serum (Gibco, USA). The chemically modified Stealth siRNA (Invitrogen) was transfected with use of Lipofectamine 2000 (Invitrogen, Carlsbad, CA, USA) according to the protocol. The sequences for control and RBP2 siRNA were 5'-CCUACAUCCCGAUCGAUGAUGUUGA-3' and 5’-CCAGCACCACCUCCUUCCUUCAUAA-3’, respectively.

**2.6 RNA extraction, RT-PCR and real-time PCR**

Extracted total RNA was purified with use of TRIzol reagent (invitrogen, USA) and reverse-transcribed by use of the RevertAid First Strand DNA Synthesis (RT) kit (Fermentas, Life Science, Canada). The primer sequences are in Table 2 **(supplement 3)**. Relative levels of mRNA were examined by use of Srby green or Taqman probe with quantitative RT-PCR (qRT-PCR) (Applied Biosystems, USA). Levels of mature miRNA were examined by TaqMan miRNA assays (Applied Biosystems, USA).

**2.7 Plasmid transfection and luciferase reporter gene assay**

PGL-p21^CIP2^ or -p27^kip1^ plasmid and pGL-TK plasmid were transfected into HCC cells. Cells were lysed 48 hr later and mixed with the dual luciferase assay reagent (Promega, USA). Psilencer-hsa-miR-212 plasmid (overexpression plasmid) and Psuper-hsa-miR-212 plasmid (inhibition plasmid) as well as the reporter plasmid containing the normal 3’ UTR with predicted hsa-miR-212 binding sites for RBP2 were constructed for luciferase assay. Cells were transfected with vector or miR-212 overexpression plasmid or co-transfected with pMIR-reporter vector containing the 3’ UTR sequence for RBP2 or mutated 3’ UTR sequence and pGL (hRluc/TK) renilla luciferase control vector. At 48 hr, luciferase activity was assayed by use of a Dual-Luciferase Reporter Assay System (Promega). Relative luciferase activity was measured as firefly luminescence normalized to that of renilla.

**2.8 Protein extraction and western blot analysis**

Total cells were lysed in lysis buffer. Cell lysates were resolved on SDS-PAGE and transferred to membranes that were incubated with the primary antibodies rabbit anti-RBP2 (Bethyl Laboratories, USA; 1:1000), mouse anti-p21^CIP2^ or -p27^kip1^ or anti-β-actin (Santa Cruz Biotechnology, USA; 1:500). Histones were extracted by a standard method by incubation with the primary antibodies rabbit anti-H3 (Abcam, USA 1:1000), anti-H3K4me2 (Abcam, USA 1:1000), or anti-H3K4me3 (Abcam, USA 1:1000).

**2.9 Clonal formation assay**

Treated cells were seeded in 6-well plates (500 cells/well) and incubated for 2 weeks, then stained with Giemsa for 10 min before methanol fixing. The number of colonies with more than 50 cells was counted.

**2.10 Senescence-associated β-galactosidase (SA-β-Gal) staining**

Cells were transfected with RBP2 siRNA for 72 hr or hsa-miR-212 plasmids for 48 hr, rinsed with phosphate buffered saline once, fixed in 3% formaldehyde for 5 min, then incubated with freshly prepared SA-β-Gal staining solution at 37ºC overnight.

**2.11** **Chromatin immunoprecipitation (ChIP)**

ChIP assay was performed according to the manufacturer’s protocol (Millipore, USA). HepG-2 cells were treated with control or RBP2 siRNA for 72 hr, cross-linked in medium containing 1% formaldehyde for 10 min at 37°C, then sonicated to obtain chromatin with DNA fragments ranging from 200 to 1000 bp. The DNA fragments bound with their corresponding elements were precipitated by incubation with primary RBP2 antibody (Abcam, USA). Protein A/G Sepharose beads ((Millipore, USA) were used to collect the protein–DNA complex, then the complex was eluted and reverse cross-linked. The phenol-chloroform method was used to extract DNA from samples with protein K treatment. Ethanol-precipitated DNA was used as a template for PCR amplification. The primer sequences used were for p27^kip1^, forward, 5’-GCTCGTCGGGGTCTGTGTCTT-3’, and reverse, 5’-GGGCCGAAGAGGTTCCTGCA-3’; p21^CIP2^, forward, 5’-GGGGCGGTTGTATATCAGG-3’, and reverse, 5’-GTGAACGCAGCACACACC-3’; and GAPDH, forward, 5’-AAAGGGCCCTGACAACTCTT-3’, and reverse, 5’-GGTGGTCCAGGGGTCTTACT-3’ as a normalization control.
