# Supplementary figures and images for: Histone Demethylase Retinoblastoma Binding Protein 2 is Overexpressed in Hepatocellular Carcinoma and Negatively Regulated by hsa-miR-212
Source: PLoS One. 2013 Jul 29;8(7):e69784. doi: 10.1371/journal.pone.0069784 (PMC3726779; doi:10.1371/journal.pone.0069784)

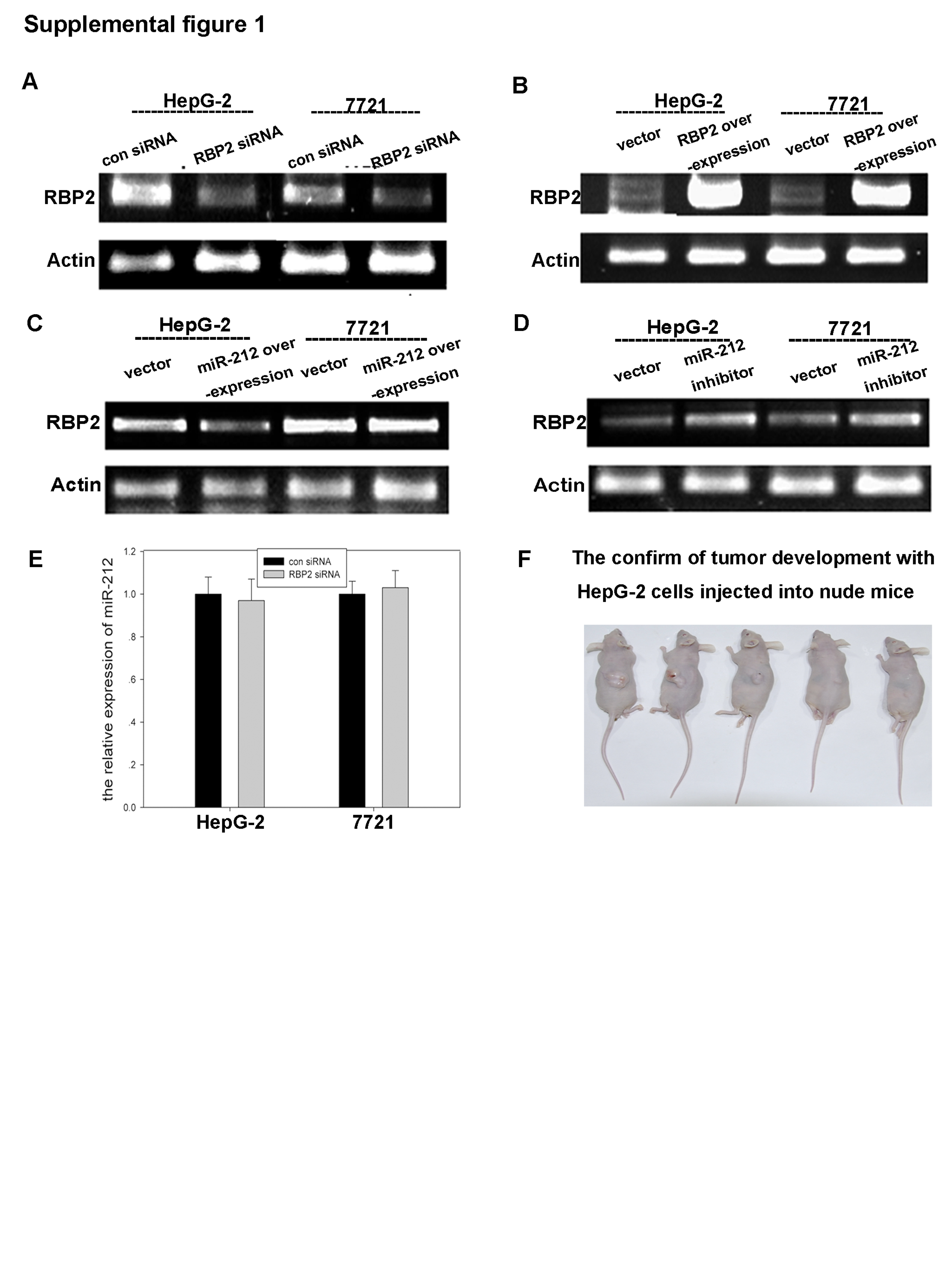

Supplement: Figure S1 — RBP2 expression was inhibited with transfection of RBP2 siRNA or enhanced with transfection of RBP2 overexpression plasmid in HCC cell lines and RBP2 was negatively regulated by hsa-miR-212 (A–B) RT-PCR analysis of the mRNA expression of RBP2 with transfection of RBP2 siRNA or RBP2 overexpression plasmid. (C–D) RT-PCR analysis of mRNA expression of RBP2 expression with transfection of hsa-miR-212 overexpression or inhibitor plasmid. (E) qRT-PCR analysis of mRNA expression of hsa-miR-212 with transfection of control or RBP2 siRNA. Data are mean±SD of biological replicates. (F) Confirmation of tumor development in mice with injection of HepG-2 cells. (TIF) [file pone.0069784.s002.tif]

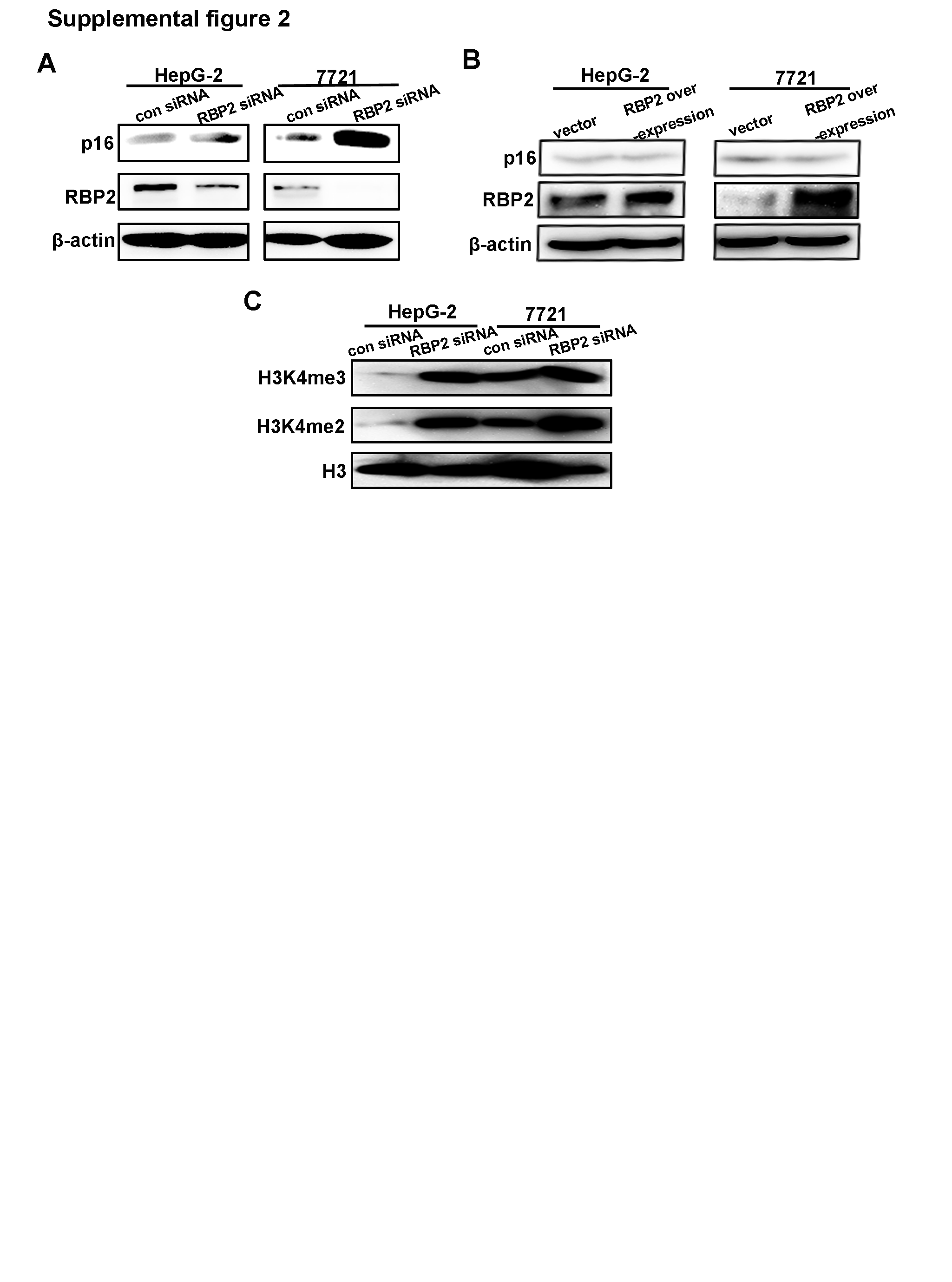

Supplement: Figure S2 — p16 was negatively regulated by RBP2 and H3K4me2 and H3K4me3 expression was significantly increased with RBP2 knockdown in HCC cell lines. (A) Western blot analysis of protein expression of p16 with RBP2 suppression and (B) RBP2 overexpression. (C) Expression of H3K4me2 and H3K4me3 expression in HCC cell lines with RBP2 knockdown. (TIF) [file pone.0069784.s003.tif]
