# Supplementary material for: Histone Demethylase Retinoblastoma Binding Protein 2 is Overexpressed in Hepatocellular Carcinoma and Negatively Regulated by hsa-miR-212
Source: PLoS One. 2013 Jul 29;8(7):e69784. doi: 10.1371/journal.pone.0069784 (PMC3726779; doi:10.1371/journal.pone.0069784)
Supplement: Table S1 — General characteristic of the patients. (DOC) [file pone.0069784.s004.doc]

| Number | **Gender** | **Age** | Diagnosis (HE) | |
| --- | --- | --- | --- | --- |
| 1 | Male | 30 | Primary HCC, right lobe |  |
| 2 | Male | 67 | HCC， high differentiated，huge，left lobe |  |
| 3 | Female | 55 | HCC, left lobe mediate differentiated，3.5cm*2.5cm*3cm |  |
| 4 | Male | 55 | HCC， right lobe |  |
| 5 | Male | 57 | HCC, 10cm*9.5cm*7cm，right lobe |  |
| 6 | Male | 56 | HCC, 7cm*7cm*5cm，right lobe |  |
| 7 | Male | 62 | HCC，right lobe |  |
| 8 | Female | 58 | HCC, high differentiated |  |
| 9 | Male | 40 | HCC, huge，right lobe |  |
| 10 | Female | 34 | HCC, diffused， metastatic，left lobe |  |
| 11 | Male | 67 | HCC，right lobe, high differentiated |  |
| 12 | Female | 31 | HCC，left lobe |  |
| 13 | Male | 42 | HCC, 7cm*3cm*2cm，right lobe |  |
| 14 | Male | 69 | HCC, coupled with cirrhosis，left lobe |  |
| 15 | Male | 56 | HCC，right lobe |  |
| 16 | Male | 40 | HCC, multiple nodular type，left lobe |  |
| 17 | Male | 52 | HCC，right lobe |  |
| 18 | Male | 40 | HCC, 6cm*3cm*3cm，right lobe |  |
| 19 | Male | 57 | HCC，left lobe |  |
| 20 | Male | 45 | HCC, nodular type，right lobe |  |

**Table 1 general characteristics of the patients**
